# Aromatherapy for the brain: Lavender's healing effect on epilepsy, depression, anxiety, migraine, and Alzheimer's disease: A review article

**DOI:** 10.1016/j.heliyon.2023.e18492

**Published:** 2023-07-20

**Authors:** Nazanin Hatami Bavarsad, Shokufeh Bagheri, Masoumeh Kourosh-Arami, Alireza Komaki

**Affiliations:** a Department of Neuroscience, School of Science and Advanced Technologies in Medicine, Hamadan University of Medical Sciences, Hamadan, Iran; b Department of Neuroscience, School of Advanced Technologies in Medicine, Iran University of Medical Sciences, Tehran, Iran

**Keywords:** Neurological disorders, Alzheimer’s disease, Lavender, Anxiety, Depression, Epilepsy, Migraine

## Abstract

Neurological diseases affect the nervous system, including the brain, spinal cord, cranial nerves, nerve roots, autonomic nervous system, neuromuscular junctions, and muscles. Herbal medicine has long been used to cure these diseases. One of these plants is lavender, which is composed of various compounds, including terpenes, such as linalool, limonene, triterpenes, linalyl acetate, alcohols, ketones, polyphenols, coumarins, cineole, and flavonoids. In this review, the literature was searched using scientific search engines and databases (Google Scholar, Science Direct, Scopus, and PubMed) for papers published between 1982 and 2020 via keywords, including review, lavender, and neurological disorders. This plant exerts its healing effect on many diseases, such as anxiety and depression through an inhibitory effect on GABA. The anti-inflammatory effects of this plant have also been documented. It improves depression by regulating glutamate receptors and inhibiting calcium channels and serotonergic factors, such as SERT. Its antiepileptic mechanism is due to an increase in the inhibitory effect of GABA and potassium current and a decrease in sodium current. Therefore, many vegetable oils are also used in herbal medicine. In this review, the healing effect of lavender on several neurological disorders, including epilepsy, depression, anxiety, migraine, and Alzheimer’s disease was investigated. All findings strongly support the traditional uses of lavender. More clinical studies are needed to investigate the effect of the plants’ pharmacological active constituents on the treatment of life-threatening diseases in humans. The limitations of this study are the low quality and the limited number of clinical studies. Different administration methods of lavender are one of the limitations of this review.

## 1 Introduction

Neurological disorders are diseases of the central and peripheral nervous systems. These disorders affect hundreds of millions of people all over the world. More than 50 million people suffer from epilepsy. Globally, there are 47.5 million people with dementia. Dementia is the major cause of Alzheimer's disease (AD) in 7.7 million new cases who develop the disease every year. The prevalence of migraine is more than 10% worldwide [1]. Patients with neurological problems may experience depression or anxiety. Symptoms of these disorders include confusion, paralysis, poor coordination, loss of sensation, seizures, muscle weakness, pain, and altered levels of consciousness. Neurological diseases are treated by the specialties of neurology and clinical neuropsychologists. Several interventions and treatments are considered preventive measures, including lifestyle changes, physiotherapy, neurorehabilitation, pain management, pharmacotherapy, operation, or following a specific diet [2,3]. In 2006, the World Health Organization (WHO), classified these neurological disorders as epilepsy, dementia, migraine, and psychiatric diseases, such as depression and anxiety.

## 2 Materials and methods

Online databases, including Scopus, ScienceDirect, Google Scholar, and PubMed were assessed for studies published between 1982 and 2020 via keywords, including review, lavender, and neurological disorders. All of the abbreviations are explained in Table 1.

**Table 1:** 

Abbreviations	
Alzheimer's disease	(AD)
World Health Organization	(WHO)
Gamma-aminobutyric acid	(GABA-A)
Amyloid precursor protein	(APP)
Amyloid β	(Aβ)
Acetylcholinesterase inhibitors	(AChEIs)
Acetylcholine	(ACh)
Transcription factor EB	(TFEB)
Generalized anxiety disorder	(GAD)
Social anxiety disorder	(SAD)
Phobias, posttraumatic stress disorder	(PTSD)
Obsessive-compulsive disorder	(OCD)
Panic disorder	(PD)
Chromogranin A	(CgA)
Serotonin reuptake inhibitors	(SSRIs)
Serotonin	(5-HT)
Voltage gated calcium channels	(VGCCs)
Norepinephrine	(NE)
Dopamine	(DA)
Histamine	(H)
Elevated plus-maze	(EPM)
Herbal Medicinal Products	(HMPC)
Electroconvulsive therapy	(ECT)
Major depressive disorder	(MDD)
European Medicines Agency	(EMA)
Serotonin transporter	(SERT)
Tricyclic antidepressants	(TCAs)
Anti-epileptic drugs	(AEDs)
Pentylenetetrazole	(PTZ)
Calcitonin gene-related peptide	(CGRP)
Linalyl acetate	(LA)
Nitric oxide synthase	(NOS)

### 2.1 Plant description

**Fig. 1:**
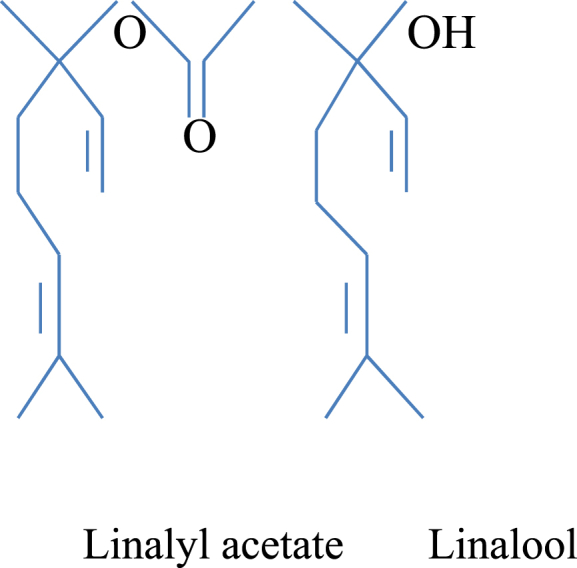
The structure of lavender containing linalool and linalyl acetate. Linalool inhibits gamma-aminobutyric acid (GABA-A) receptors.

Lavender is a plant of the mint or Lamiaceae family [4]. This family has different types and species, including *Lavandula angustifolia, Lavandula angustifolia*, *Lavandula hybrida* Rev, *Melissa officinali*s L., *Lavandula stoechas*, *Lavandula latifolia*, and *Lavandula x intermedia* (a cross between *L. latifolia* and *L. angustifolia*) [4,5]. Lavender contains 100 different combinations, including terpenes, like linalool, limonene, triterpenes, linalyl acetate, alcohols, ketones, polyphenols, coumarins, cineole, and flavonoids [4] (Fig. 1). *L. angustifolia*, *L. latifolia,* and *L. Intermedia* are used to extract essential oils [6]. Linalool in this plant has an inhibitory effect through gamma-aminobutyric acid (GABA-A) receptors. This effect inhibits the limbic system and reduces anxiety [7,8].

Essential oils are abundant and volatile compounds obtained from plant parts, like leaves, seeds, and flowers through different methods, such as distillation [5]. The flowers of this plant are used in decoration in ceremonies and its oil is also used in the cosmetics and perfume industry [9,10]. This plant, especially as an essential oil, has various applications in medicine to treat psychological diseases, such as calming the nervous system, particularly in cases with depression, anxiety, and sleep disorders [11,12]. Its positive effects on the nervous system are related to the impact of its volatile compounds on the hippocampus and amygdala [13].

Aromatherapy using its oil, especially in anxiety and sleep disorders, has been found with very promising results [14]. Lavender has been employed using various methods in different studies, including inhalation, massage therapy, combined massage therapy with aromatherapy, and oral administration [10] (Table 2). It has also been used for the treatment of bloating and for wound healing (as an antibacterial and antifungal agent) and the treatment of insect bites. Lavender has analgesic, anti-inflammatory, and antispasmodic effects, as well [11,12].

**Table 2:** The doses, intervention durations, therapeutic response, and mechanism of action of lavender.

Author(s)	Study year	Study design	Treatment	Treatment duration	Therapeutic response and mechanism of action of lavender	Diseases
Lin et al.	2007	Cross-over randomized trial	Lavender inhalation Sunflower inhalation	3 weeks	Alleviated agitation in cases with dementia	AD
Hancianu et al.	2013	Experimental study	Lavender oils	7 days	Neuroprotective, antioxidant, and antiapoptotic effects	Dementia
Faixova et al.	2008	Clinical study	Rosemary oil (*Rosmarinus officinalis* L., Labiatae, 1 ml)	20 min	Relieved depression and stress	Depression and stress
Azizi et al.	2012	Experimental study	Thymol or carvacrol (0.5, 1, or 2 mg/kg)	14 days	Improvement in cognitive impairments by thymol and carvacrol induced by Aβ or cholinergic hypofunction	Dementia
Karan NB	2019	Randomized clinical trial	Lavender oil	3 min	Lavender oil has strong anxiolytic effects	Anxiety
Komaki et al.	2014	Experimental study	Linalyl acetate (50 and 100 mg/kg)	30 min	Anti-anxiety effects via modulating voltage-dependent calcium channels and glutamatergic transmission	Anxiety
Schuwald et al.	2013	Clinical trial	Silexan (1 and 30 mg/kg/day), 0.2% agar suspension (10 ml/kg), diazepam (2.5 mg/kg), or pregabalin (100 mg/kg)	3 days	Anxiolytic effects by modulating voltage-dependent calcium channels	Anxiety
Ebrahimi et al.	2022	Clinical trial	Lavender and chamomile essential oils	30 days	Decreased depression, anxiety, and stress	Anxiety and Depression
Xiong et al.	2017	Clinical trial	50 μL of lavender [*Lavandula angustifolia*], sweet orange [*Citrus sinensis*], and bergamot (*Citrus bergamia*) oils	8 weeks	Decreased depression by an aromatherapy massage and aromatherapy inhalation	Depression
Linck et al.	2010	Experimental study	Linalool and Diazepam (0.5 and 1.0 mg/kg)	60 min	Anxiolytic effects increased social interaction and decreased aggressive behavior in rats	Anxiety
de Almeida et al.	2012	Experimental study	Limonene epoxide at the doses of 25, 50, and 75 mg/kg (i.p.)	14 days	Anxiolytic-like effect on mice	Anxiety
Effati-Daryani et al.	2015	Randomized placebo-controlled trial	Lavender cream	8 weeks	Reduction in anxiety, stress, and depression	Anxiety and Depression
Tayebi et al.	2015	Clinical trial	Lavender essential oil	4 weeks	The scores of depression and stress significantly decreased but this reduction was not significant for anxiety scores.	Anxiety and Depression
Bazrafshan et al.	2020	Randomized clinical trial	Inhaled lavender	4 weeks	Reduction in anxiety-depression score Blockage of the serotonin transporter (SERT) and antagonism effects on the N-methyl-d-aspartate receptors (NMDA receptors)	Anxiety and Depression
Shin et al.	2018	Experimental study	Linalyl acetate (10 or 100 mg/kg) or metformin (500 mg/kg)	7 days	Linalyl acetate can be efficient in diabetic patients with cardiovascular disease or chronic stress LA restored serum nitrite levels, acetylcholine-induced vasorelaxation, blood pressure, heart rate, and AMP-activated protein kinase	Chronic stress
Zamanifar et al.	2020	Randomized controlled clinical trial	1.5% chamomile-lavender	20 min	Reduced anxiety of nurses in the clinical setting	Anxiety
Karimzadeh et al.	2021	Randomized controlled clinical trial	5 drops of lavender	30 min	Improved the state of anxiety and agitation management in the ICU setting	Anxiety
Emami-Moghadam et al.	2022	Randomized controlled clinical trial	Lavender	30 min	Reduced ECT-related anxiety in depressed patients	Anxiety
Araj-Khodaei et al.	2020	Randomized controlled clinical trial	*L. angustifolia* (2 g) or fluoxetine (20 mg)	8 weeks	Anti-depressant effect	Depression
Firoozeei et al.	2020	Randomized controlled clinical trial	Lavender-dodder herbal syrup (5 ml)	3 weeks	Treated major depressive disorder with anxious distress	Depression
Chen et al.	2021	Randomized controlled clinical trial	100% *Lavandula angustifolia* (0.1 ml) + 100% *Citrus bergamia* (0.1 ml)	28 days	Improved headache-related quality of life among nurses working in the emergency and critical care units	Migraine
Rakotosaona et al.	2016	Experimental study	C. madagascariensis (CMEO)- (0.4 and 0.8 ml/kg bw)	4 weeks	Anticonvulsant effects	Epilepsy
Rafie et al.	2016	Randomized controlled clinical trial	Lavender	3 months	Reduction in frequency and severity of migraine	Migraine

## 3 The effects of lavender on neurological/neuronal disorders

### 3.1 Lavender and Alzheimer’s disease (AD)

AD is a neurodegenerative multifarious disease [15]. It is the most common neurodegenerative disease [5]. The combination of genetic and environmental factors plays a role in the development of AD. It has familial and sporadic types and there is early- or late-onset AD [16,17]. Mutation in specific genes, such as presenilin I and II, amyloid precursor protein (APP), and apolipoprotein E is associated with the pathophysiology of this disorder [18]. Dementia is observed in 5% of people over the age of 65 and most dementia patients have psychological and behavioral symptoms [19]. AD as a cognitive disorder is the most common form of dementia and accounts for 2% of its types. Several hypotheses, such as senile plaques and neurofibrillary tangles have been suggested as the pathobiology of the disorder. The plaques are formed from amyloid-β (Aβ) peptides, and neurofibrillary tangles are created from hyper-phosphorylated tau proteins [20,21]. There are several other theories, like the cholinergic hypothesis, oxidative stress hypothesis, mitochondrial cascade hypothesis, and inflammation hypothesis. When the balance between oxidants and antioxidants is disturbed, free radicals are produced that damage neurons. The accumulation of α-synuclein and neurodegenerative progression is guided by the proinflammation stage. Besides, a few viruses may have a role as stimulators and generate a cross-autoimmune response for α-synuclein [22]. Cholinesterase inhibitor prevents the breakdown of acetylcholine (ACh), which is an important neurotransmitter in memory [23]. Acetylcholinesterase inhibitors (AChEIs) having antioxidant potential can enhance learning and memory [24]. Piperazine-tethered biphenyl-3-oxo-1,2,4-triazine derivatives were synthesized as a non-competitive acetylcholinesterase inhibitor [25]. During the process of aerobic respiration, radicals are produced and attack the cell. Oxidative stress plays a very important role in the pathobiology of AD. Increasing age and AD cause an increase in the production of free radicals, which leads to subsequent oxidative damage. Another molecular target, namely transcription factor EB (TFEB), has been explored globally to treat neurodegenerative disorders. This TFEB is a key regulator of autophagy and the lysosomal biogenesis pathway [26]. Herbal remedies play a crucial role in the progression of medicine, and many advanced drugs have already been developed. Many studies have endorsed practicing herbal remedies with phytoconstituents for healing AD [27]. Essential oils rich in antioxidants fight free radicals that cause oxidative stress [23,[28], [29], [30]]. Licensed and marketed drugs for AD include cholinesterase inhibitors, like rivastigmine, galantamine, and donepezil. Also, N-AChE was the first drug approved in 1993; however, its use was discontinued due to many side effects [31,32]. Essential oils of vegetables affect brain function due to crossing the blood-brain barrier [23]. The aroma of vegetable oils used through inhalation or massage affects the limbic system after penetrating into the blood system [33].

**Fig. 2:**
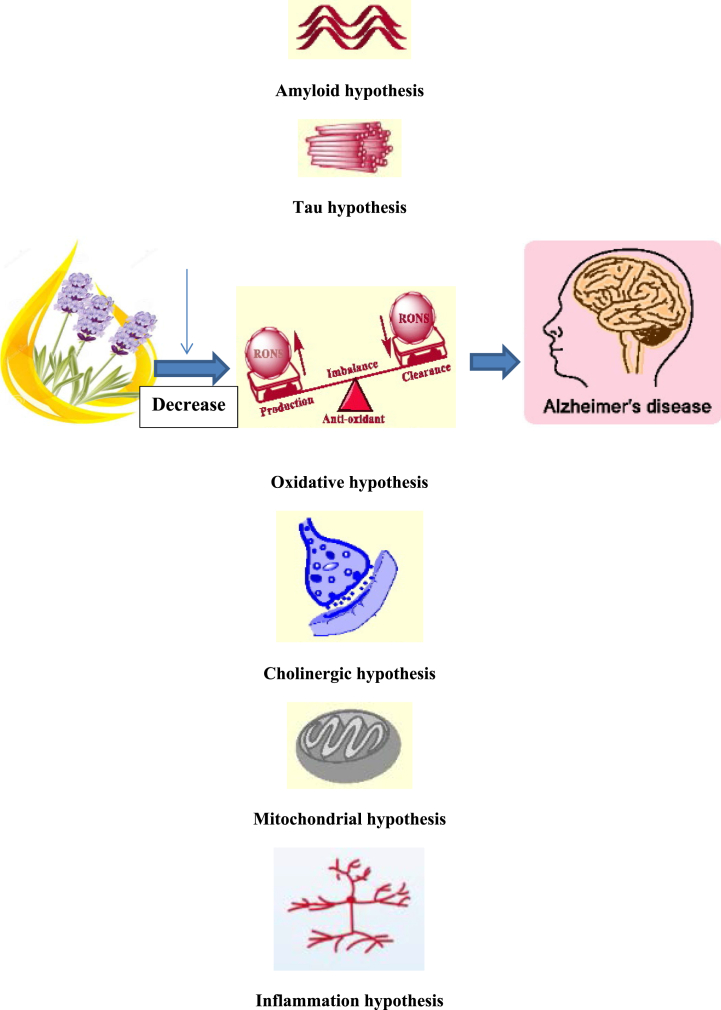
Pathophysiology of Alzheimer's Disease (AD); antioxidant, anti-inflammatory, and anti-cholinergic effects of essential oils.

Essential oils used through inhalation or massage directly affect cognition, but most human and animal studies have preferred the inhalation method to study AD. Oils entering the blood probably reach the limbic system through the nose and lung mucus and affect the olfactory nerve [33]. The effect of essential oils is related to the cholinergic hypothesis and oxidative stress hypothesis. A decrease in Ach and the loss of cholinergic neurons impair cognitive function and memory. The oxidative stress hypothesis is related to the factors causing AD, such as Aβ-induced neuronal loss, tau protein pathology, mitochondria dysfunction, and disturbance in metal homeostasis [[34], [35], [36]]. Essential vegetable oils have received considerable attention due to their antioxidant and anti-amyloid, and cholinesterase inhibitory properties [37] (Fig. 2). In 2007, Lin et al. showed the effect of essential oils obtained from *L. angustifolia* on agitation in patients with dementia and AD [38]. Hancianu et al. in 2013 assessed the effect of *L. angustifolia*, *L. Angustifolia* Mill., and hybrid lavender and showed their significant antioxidant and antiapoptotic potentials and their ability to increase the level of immune system antioxidant enzymes in scopolamine-induced dementia rat models [39]. Also, it has been shown that essential oils of *L. angustifolia* Mill. has a long-term effect on attention [40]. *Rosmarinus officinalis* L. has strong antioxidant compounds that reduce oxidative stress as one of the causes of AD [5] (Fig. 2). The combination of essential oils derived from *R. officinalis* can improve memory in dogs and rats [41]. *M. officinalis* L. is also a strong antioxidant and reduces oxidative stress and protects the brain against oxidative damage [23,42]. Essential oils obtained from L. angustifolia are rich in thymol. Azizi et al. demonstrated that different doses of thymol or carvacrol (0.5, 1, or 2 mg/kg) can improve cognitive function in animals with impaired memory induced by the injection of scopolamine and Ab25–35 peptide. The beneficial effects observed in these models may be due to the anticholinesterase, antioxidant, and anti-inflammatory activities of thymol and carvacrol [43,44]. The antioxidant and antiapoptotic effect of *L. angustifolia*, *L. Angustifolia* Mill., and hybrid lavender has been reported in scopolamine-induced dementia in rats. Essential oils obtained from *L. angustifolia* and *R. officinalis* improved cognitive functions and memory in animal models.

### 3.2 Lavender and anxiety

Anxiety is one of the psychiatric disorders and women are nearly twice as likely to experience anxiety as men [45]. This disorder is associated with excessive fear related to acute dysfunction of the autonomic system and behavioral disturbance [46]. It triggers the release of hormones, such as cortisol, noradrenaline, and adrenaline by affecting the sympathetic nervous system [47]. Anxiety is a vague, unpleasant, and diffuse feeling of anxiety associated with physical symptoms, such as sweating and high blood pressure [48]. About 4% of the world's population has one or more than one anxiety disorders [49]. The prevalence of fear and anxiety in western societies is about 20–30%. The disease imposes a significant economic burden on society [50].

Different types of anxiety disorders are generalized anxiety disorder (GAD), social anxiety disorder (SAD), phobias, posttraumatic stress disorder (PTSD), obsessive-compulsive disorder (OCD), and panic disorder (PD) [50]. In addition to blood pressure and heart rate, stress markers, including cortisol and salivary chromogranin A (CgA), are used to assess anxiety [51]. Anxiety is treated with psychotherapy and medication but patients with more severe anxiety disorders may develop depression and physical illnesses and are prone to substance abuse [52]. Anxiety medications, such as benzodiazepines and selective serotonin reuptake inhibitors (SSRIs) have side effects, like amnesia, sedation, and diminished concentration [53]. Therefore, there is a need for more effective drugs with fewer side effects [54]. Also, even patients using drugs, psychotherapy, and brain stimulation therapies show little or no improvement, which indicates the need for newer methods to treat this disorder [50,55,56].

Based on studies performed on normal people and people with anxiety, some chemicals and neurotransmitters have been shown to be involved in anxiety [57]. Neurotransmitters involved in this disorder include serotonin (5-HT), norepinephrine (NE), GABA, dopamine (DA), histamine (H), and Ach [57,58]. The sedative effects of lavender oil inhalation have been extensively studied on animals [7]. The acute effects of hydro-alcoholic extract of lavender (25, 50, or 100 mg/kg, intraperitoneally) on anxiety-like behavior in rats were demonstrated using an elevated plus-maze (EPM) test. Lavender extract showed an anxiolytic effect similar to that of low-dose diazepam [59]. Various studies have shown the effect of lavender oil in the form of creams and perfumes on the treatment of anxiety and its effect on anxiety is similar to chlordiazepoxide and is more effective than lorazepam [60]. In different studies, the positive effects of lavender using various methods, such as massage and inhalation during labor, have been shown [61]. Siloxane is a standardized form of lavender essential oil collected from the distillation of the fresh *L. angustifolia* Miller flowers [62]. Silexan acts through the potent inhibition of voltage-dependent calcium channels in brain areas, such as the hippocampal neurons [63]. The Committee on Herbal Medicinal Products (HMPC) of the European Medicines Agency (EMA) has accepted the use of oral lavender essential oil for the treatment of mental stress, and it is licensed in 14 countries around the world. Lavender oil has been shown to inhibit voltage-dependent calcium channels (VOCCs) in synaptosomes, primary hippocampal neurons, and stably overexpressing cell lines in a similar range as pregabalin. This inhibition is mainly mediated via N-type and P/Q-type VOCCs. However, further investigations are needed to assess the long-term effects of lavender use [4,64]. Lavender is one of the most effective and healthy choices for treating anxiety [8]. Essential oils of lavender increased happiness hormones even by ten times [65].

*L. angustifolia* (also named *L. vera* or *L. officinalis*), which is the most widely used species of lavender, has important compounds, including linalyl acetate and linalool. These compounds are responsible for the medicinal effects of the plant, including its sedative effects [4]. Linalool in lavender has an inhibitory effect on the GABA-A receptors, and autonomic and limbic systems that lower blood pressure [7,63].

Lavender essential oils exert their calming effect through various proposed mechanisms, such as GABA receptor inhibition, the reaction of linalool with glutamatergic NMDA receptors, inhibition of serotonin transporter (SERT), antagonizing the NMDA receptor, inhibiting tension-dependent calcium channels, and affecting the 5HT-1A receptor in specific brain areas, such as temporal gyrus [4,64,[66], [67], [68]]. The results of a study showed that the interventions using music therapy and aromatherapy with chamomile-lavender essential oil could reduce the anxiety of nurses in clinical settings [69]. In 2021, the positive effects of lavender aromatherapy and *Citrus aurantium* aromatherapy on reducing the anxiety of patients were documented [70]. Inhalation with 1.5% lavender and chamomile essential oils for 30 nights decreased depression, anxiety, and stress levels in community-dwelling older adults [71]. Aromatherapy with inhaled lavender essential oil and breathing exercises can be considered an effective intervention to reduce electroconvulsive therapy (ECT)-related anxiety in depressed patients [72]. Aromatherapy with lavender treated anxiety and depression in patients. This herb has a sedative effect through the inhibition of GABA receptor, inhibition of SERT, antagonizing the NMDA receptors, inhibition of tension-dependent calcium channels, and affecting the 5HT-1A receptors in specific brain areas, such as temporal gyrus.

### 3.3 Lavender and depression

Depressive disorder is one of the cognitive disorders [73]. Depression is a major mental health problem in the world, affecting more than 350 million people [74]. In most cases, depression is the result of or associated with chronic illnesses or mood disorders [75]. Decreased concentration, anxiety, low mood, and anhedonia are some of the symptoms of this disorder [76].

The most important type of depression is major depression, which has symptoms, like insomnia, fatigue, the lack of pleasure, feeling guilty, suicidal thoughts or attempts to suicide, weight loss, or loss of appetite. Tricyclic antidepressants (TCAs), like imipramine and amitriptyline, are still prescribed to treat depression. One of the most common antidepressants is fluoxetine as an SSRI [77]. Trazodone and amoxapine are also used for the treatment of this disorder [78]. Nearly 50% of depressed patients respond positively to pharmacological treatment [79]. It has recently been shown that exercise as a non-pharmacological method is effective in the treatment of this disorder [80]. Aromatherapy by massage and inhalation can alleviate the symptoms of depression [81].

Depression is one of the most common emotional problems, especially in women. Because of the side effects of antidepressants, the use of alternative and complementary therapies has been considered [82,83]. Different psychotherapeutic approaches are used to treat depression, which are not followed by the patient because they are time-consuming and costly [[84], [85], [86]]. Although drugs, like SSRI, are effective in treating depression, they are associated with side effects, like developing neuropsychiatric conditions, such as sleep disorders and sexual dysfunction. Thus, there is a need to develop new treatments, especially for prevention and the treatment of a milder type of disorder [87,88]. Aromatherapy is effective in improving mental and physical function [89]. Also, this method has few side effects and is not expensive [90]. Aromatherapy has been reported to be effective in reducing depression in postmenopausal women, cases with major depressive disorder (MDD), postpartum women, and patients with cancer [81]. In a test conducted in 2014, it was found that aromatherapy can be effective in relieving depression, pain, and stress in adults [91]. In Europe, lavender oil is traditionally used to reduce stress and anxiety [88]. One of the best-selling herbal oils for depression is lavender essential oils [92]. Monoterpenes, such as linalool and linalyl acetate in lavender essential oil, are responsible for its anti-depressant properties [93,94].

**Fig. 3:**
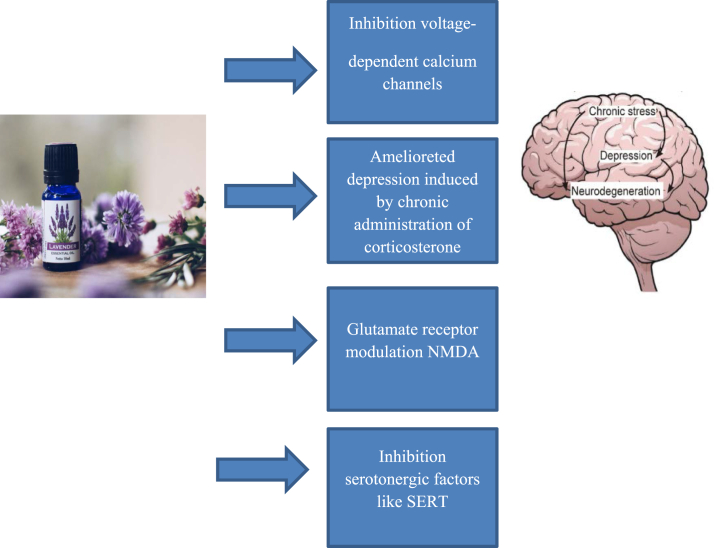
Aromatherapy by lavender treats depression by inhibiting voltage-dependent calcium channels and serotonergic factors and modulating NMDA receptors.

The antidepressant effect of lavender is related to its effect on the modulation of glutamate NMDA-receptor and SERT [88]. Linalool in lavender can react with the glutaminergic system and NMDA receptors [95,96] (Fig. 3). The hydroxyl group of linalool also appears to be responsible for its effect on the serotonin transporter (SERT). Administration of a mixture of cis and trans (+)-limonene epoxide has been shown to exert an anxiolytic-like effect in mice. In the open field test, intraperitoneal administration of (+)-limonene epoxide at doses of 25, 50, and 75 mg/kg significantly reduced the number of crossings, grooming, and rearing [97].

In a study in 2013, it was found that the essential oils of this plant can inhibit voltage-dependent calcium channels in brain areas, such as primary hippocampal neurons [94]. Essential oils of the plants, such as lavender, inhibit serotonergic factors, like SERT, which indicates their antidepressant effects. In a study, M. officinalis (2 g), L. angustifolia (2 g), or fluoxetine (20 mg) were administered to participants, and their effects were assessed in weeks 0, 2, 4, and 8 using the Hamilton Rating Scale for Depression (HAM-D). Both M. officinalis and L. angustifolia showed similar effects to fluoxetine in treating mild to moderate depression [98,99] (Fig. 3). Also, *L. angustiifolia* has antidepressant, sedative, and antidepressant effects because of the compounds, such as linalool, linalyl acetate, flavonoid, local anesthetic tannins, ocimene, caryophyllene oxide, and coumarin [[100], [101], [102], [103]]. Lavender with compounds, such as linalool and linalyl acetate was effective in improving mood in pregnant women used by inhalation in a short period of time [104]. The therapeutic effects of lavender cream, either used with foot-bath or alone (2 g applied every night for two months), on the mental health of pregnant women with depression and stress have been reported [105]. Also, its therapeutic effects on hemodialysis patients suffering from stress and depression have been shown [106]. In a study conducted in 2020 in Iran, the mean score of depression and anxiety in the lavender group showed a decrease compared with the controls [107]. The consumption of lavender herbal tea has been shown to reduce depression and anxiety scores [108]. Additionally, taking 5 mL of lavender-dodder herbal syrup every 12 h has been found to be an effective and well-tolerated supplement for treating major depressive disorder (MDD) with anxious distress [109]. In a similar clinical trial on the effect of inhalation of lavender, aromatherapy with lavender essential oils decreased stress, depression, and anxiety in older adults compared to the control group [71].

Aromatherapy is effective in reducing depression in postmenopausal women, cases with major depressive disorder, postpartum women, and patients with cancer. Lavender can modulate the glutaminergic system and NMDA receptors.

### 3.4 Effect of lavender oil on epilepsy

**Fig. 4:**
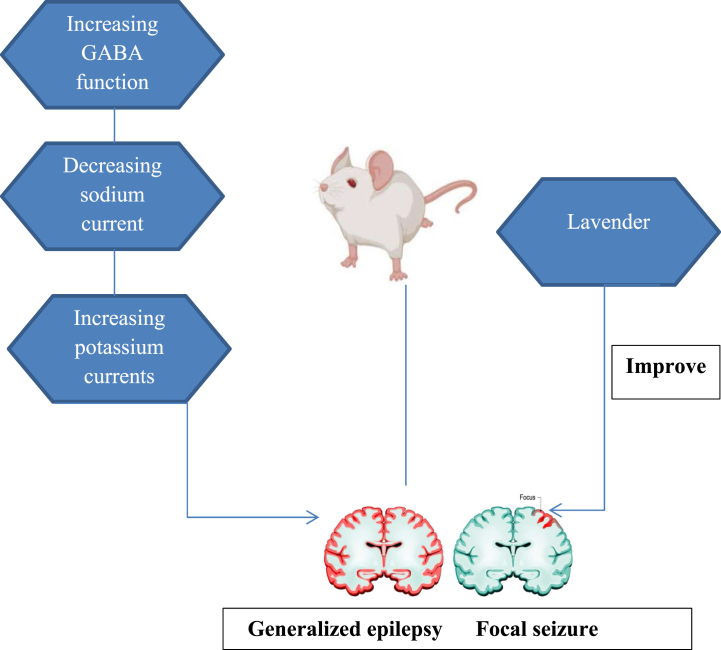
Lavender exerts antiepileptic effects by increasing potassium current and GABA function and reducing sodium current.

Approximately 20–30% of patients with epilepsy have drug-resistant seizures [110,111]. Also, seizure recurrence is observed in 5% of people worldwide, and 35% of people have uncontrolled epilepsy [112]. Proper diagnosis of epilepsy syndrome is essential, and when refractory epilepsy is confirmed, surgery is regarded for patients [[113], [114], [115]]. Surgery is used selectively for patients with treatment-resistant focal seizures [116,117]. Other treatments for epilepsy are underused vagal nerve stimulation [112] or closed-loop cortical stimulation [118]. New-generation anti-epileptic drugs (AEDs) have side effects, including psychological complications, like depression, anxiety, and cognitive deficits [110]. However, these AEDs have fewer side effects in comparison with older-generation AEDs [119]. Natural products used in traditional medicine are powerful sources of new AEDs. They have been primary treatments for patients with epilepsy because of their availability. Also, Ekstein in 2010, confirmed that new drug therapies are more cost-effective for patients [119]. Lifestyle changes can prevent seizure precipitation in teenagers with idiopathic generalized epilepsy [112]. Strong anticonvulsive effects of lavender and other essential oils high in linalool have been demonstrated in animal models of seizure [120]. Linalool is known as a monoterpene alcohol that strengthens GABAA function in mammalian electrophysiology experiments [121]. Linalool oxide, linalyl acetate, 8-oxo linalyl acetate, 8-carboxy linalyl acetate, and 8-oxo linalool are linalool derivatives and metabolites. Also, these metabolites play a role in GABA-A function [121,122]. In snail neurons, linalool inhibits sodium channels and increases potassium currents (Fig. 4). The lower concentration of linalool has a suppressive effect on spontaneous activity and PTZ-induced epileptiform activity. A higher concentration of linalool can induce epileptiform activities, reversible by calcium channel blockers [123]. The administration of Cinnamosma madagascariensis (0.4 and 0.8 mL/kg bw), Zhumeria majdae, and Citrus aurantium blossom oils has been found to decrease convulsions in animals treated with PTZ. Furthermore, these oils have been shown to increase latency and survival [[124], [125], [126]]. Inhaled lavender essential oil (1 mL) administrated 15 min before pentylenetetrazole (PTZ) treatment, could prevent all convulsions in 100% of the animals leading to a 100% survival rate. The animals of the control group receiving PTZ were found with seizures and at this dose, there was a 100% mortality rate [127]. Lavender increases GABAA function and potassium current. Inhaled lavender essential oil before PTZ treatment could prevent all convulsions in animals.

### 3.5 Effect of lavender on migraine

Migraine is a chronic disease and according to the World Health Organization, it is the sixth most disabling disease worldwide among all neurological disorders (G. B. D. Neurological Disorders Collaborator Group, 2017). It has been reported that 17% of women and 6% of men have migraine [128]. Nausea, vomiting, and sensitivity to light and sound are some of its symptoms. Migraine headaches with moderate to severe intensity, start on one side of the head. They occur through hypothalamic disturbance that changes blood flow through intra- and extra-cranial blood vessels by a trigeminovascular reflex [129,130]. Different mental, environmental, and genetic factors are involved in the development of the disease [131,132]. In China, the prevalence rate of migraine was about 9.3% in 2012 [132]. This neurological disorder is associated with hormonal changes. After puberty, the incidence of migraine increases, whereas its prevalence decreases during pregnancy [133,134]. There is an increase in plasma levels of calcitonin gene-related peptide (CGRP), a strong vasodilator and marker of trigeminal inflammation, in patients with migraine [135]. Different factors, like nutritional habits, hormonal diseases, digestive disorders, autoimmune disorders, structural imbalances, mental stress, and lifestyle are involved in the pathology of migraine [136]. Venlafaxine, beta-blockers, valproate, topiramate, amitriptyline, flunarizine, magnesium, gabapentin, and botulinum toxin type A are used to treat migraine [137]. The headache frequency and severity are prevented by supplements and medicinal herbs [138,139]. The effectiveness of traditional medicine and medicinal herbs has been demonstrated worldwide [140]. Pharmacotherapy, massage therapy, acupuncture therapy, and aromatherapy are used for the treatment of migraine. The best treatment for relieving the symptoms of the disease is aromatherapy. Traditional Chinese Medicine Aromatherapy is a monograph that indicates the efficacy of aromatic plants and their application in the treatment of different diseases [141]. Based on a single-blind study assessing the effectiveness of the topical usage of lavender oil on migraine, 47 patients with migraine at the beginning of attacks, were administrated with two to three drops of a placebo solution (liquid paraffin) or lavender oil rubbed onto their upper lip followed by inhalation the vapor for 15 min [142]. The procedure was employed for six consecutive migraine attacks. According to the results obtained from the 129 migraine attacks through the research period, the lavender group had a 3.6-point decrease in rating severity (on a 10-point rating scale), which was significantly more compared to the 1.6-point decrease in rating severity in the placebo group (obtained data from 68 attacks). The lavender group (71%) also showed a higher percentage of total or partial responders than the placebo group (47%). In addition, 74% of the subjects in the lavender group showed an improvement in migraine symptoms (vomiting, nausea, photophobia, and phonophobia) in comparison with only 58% in the placebo group. In animal models, lavender caused a reduction in inflammation and neuropathic pain by affecting peripheral and central opioid and cannabinoid 2 receptors [143]. In another animal research, after renal ischemia/reperfusion injury, lavender oil significantly improved antioxidant enzyme activity, decreased lipid peroxidation, and markedly reduced concentration of the cytokines, tumor necrosis factor-α, and interleukin-1β [144]. Also, the effect of lavender as a prophylactic therapy was investigetad for migraine in a randomized controlled clinical trial. In a three-month trial, the group that received lavender showed a decrease in the frequency and severity of migraine attacks. Additionally, lavender oil was found to be effective in preventing migraines [140]. Studies on human cell lines have indicated the efficacy of estrogenic and antiandrogenic properties of the lavender oil and it can be used in adults with an obvious allergy to lavender [145].

**Fig. 5:**
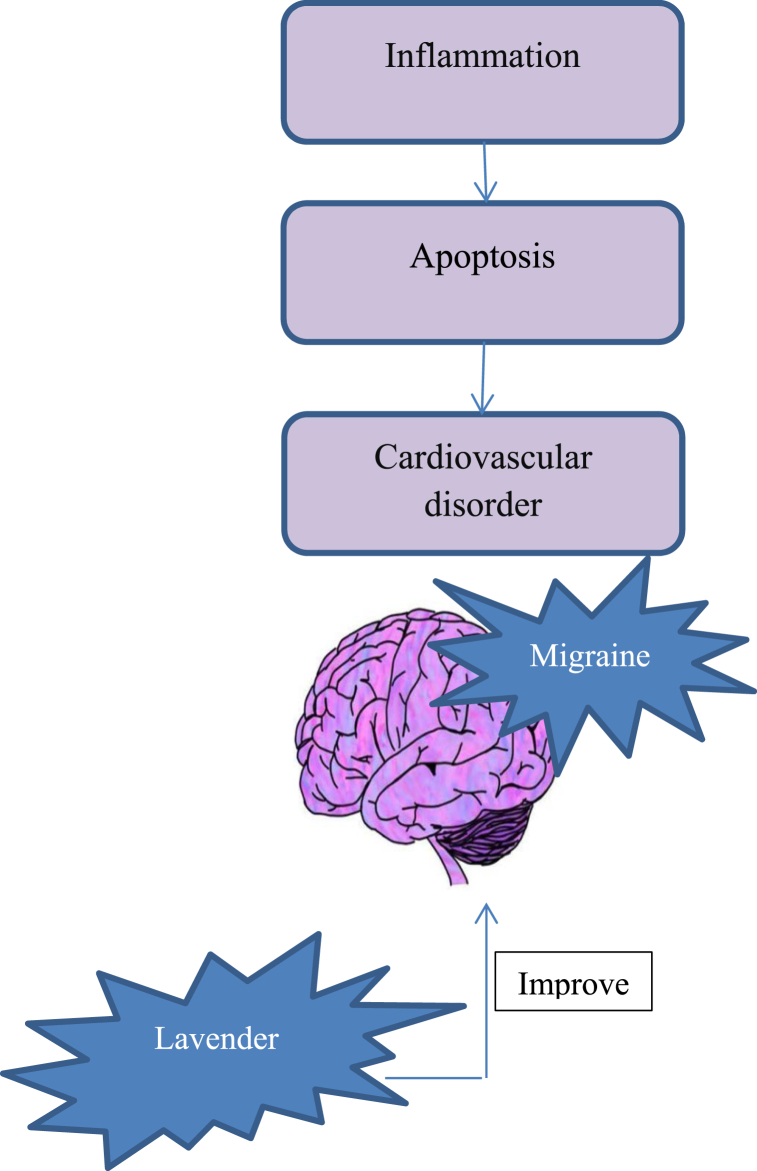
Linalyl acetate (LA) in lavender oil exerts anti-migraine effects due to its anti-inflammatory effect. Also, isolated aortic vasodilation is caused by LA.

Linalyl acetate (LA), available in lavender oil, exerts its anti-inflammatory effects by inhibiting the NF-κB activation. According to the results reported by Koto et al. (2006), rabbit carotid arteries were dilated by lavender [146]. Arginine nitrate, as a nitric oxide synthase (NOS) inhibitor, and 1H-[1,2,4] oxadiazole [4,3-α] quinoxaline-1-one, as the guanylyl cyclase inhibitor, have relaxation effects [141]. Shin et al. (2018) also found that in diabetic rats, the isolated aortic vasodilation caused by stress from ACh increased following an intraperitoneal injection of a low concentration of LA [147] (Fig. 5). In addition, by an increase in the concentration of LA, ACh-related vasodilation approximately reached the control levels. Accordingly, LA may have an anti-migraine effect via inhibiting neurogenic inflammation and balancing vasomotor impairments. Therefore, more studies on plant essential oils are needed to develop novel anti-migraine agents [141]. A study found that using a 1:1 combination of essential oils from Lavandula angustifolia and Citrus bergamia (0.1 mL of 100% Lavandula angustifolia +0.1 mL of 100% Citrus bergamia) for 28 days effectively improved the quality of life, particularly among nurses working in emergency and critical care units during the intervention period [148]. Lavender oil significantly improved antioxidant enzyme activity, reduced lipid peroxidation, decreased the concentration of the cytokines, tumor necrosis factor-α, and interleukin-1β, and improved migraine in human, animal, and cell studies.

## 4 Summary

**Fig. 6:**
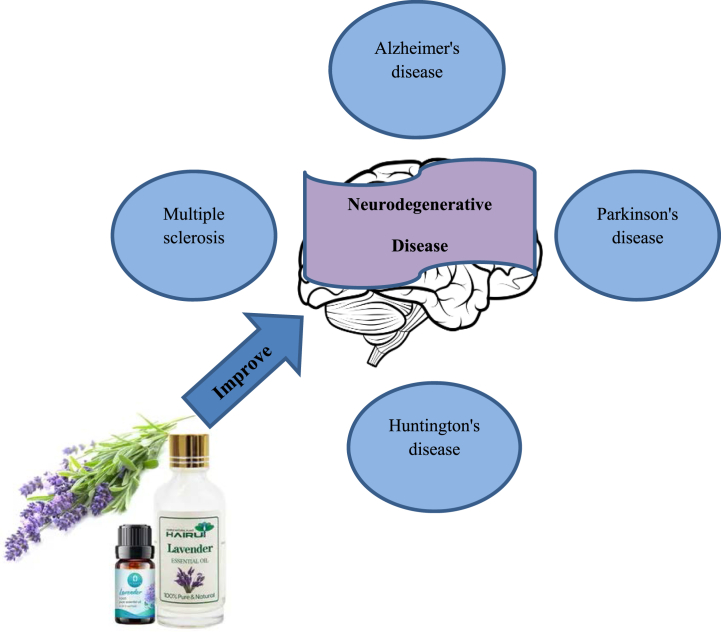
Lavender may have a beneficial effect on neurodegenerative diseases such Alzheimer's disease.

Lavender has several therapeutic effects, ranging from inducing relaxation to treating parasitic infections, burns, insect bites, and spasms. There is growing evidence suggesting that lavender oil may be an effective medicament in the treatment of several neurological disorders. Several animal and human investigations have reported the anxiolytic, mood stabilizer, sedative, analgesic, anticonvulsive, and neuroprotective properties of lavender. These studies raised the possibility of the revival of lavender's therapeutic efficacy in neurological disorders. In this paper, we reviewed the current experimental and clinical state of knowledge about the effect of lavender on the nervous system [149]. Lavender is effective on neurodegenerative diseases [150]. There is some evidence to suggest that lavender may have a beneficial effect on neurodegenerative diseases such Alzheimer's disease (Fig. 6). Although there is considerable debate as to whether lavender species have the significant clinical potential either alone or as an adjunct to other substances, many human studies support its effectiveness in different neurological and psychological disorders. Lavender was used predominantly by oral administration, aromatherapy, or massage in several clinical studies with several advantages. In addition to psychological effects, aromatherapy is thought to be therapeutically effective due to the physiological effects of inhaled volatile compounds. It is believed that inhaled lavender acts on the limbic system, particularly the amygdala, and hippocampus [151]. Linalool and linalyl acetate are rapidly absorbed through the skin after topical application with massage and can cause central nervous system depression [152].

The recent rise in popularity of alternative medicine and natural products has increased interest in lavender and its essential oils as potential natural remedies [153]. This review may be useful in increasing our knowledge of lavender's pharmacological effects and improving our future experimental and clinical research plans. Although lavender may have a significant clinical potential either alone or as adjuvant therapy in different disorders, due to some issues, such as methodological inadequacies, small sample sizes, short duration of lavender application, lack of information regarding appropriate dose, variation between efficacy and effectiveness in trials, variability of administration methods, the absence of a placebo comparator, or the lack of control groups in the conducted studies, more standard experiments and studies are needed to confirm the beneficial effect of lavender on the neurological disorders [154]. Methodological and oil identification problems have also hampered the evaluation of the therapeutic significance of some research on lavender. The dried lavender flowers used in some trials were sourced from a local herb store [155]. Although taxonomic identification was confirmed in these studies [156], without quantification of key constituents, the quality of the herbal product may be questionable. Although some studies have defined the contents of lavender, it is imperative that all future clinical studies specify the exact derivatives of the oils used in the study and preferably include the liquid profile or percentage of the main constituents. In addition, several factors, such as temperature, skin type and quality, and the size of the area being treated, which may affect the level and rate of lavender absorption after massage or aromatherapy, have not been considered in several investigations. Many discreet compounds in lavender oil have shown a myriad of potential therapeutic effects, and researchers are still looking for new treatments for various diseases [153]. Lavender has healing effects through inhibitory effects on GABA. The anti-inflammatory effects of this plant have also been proven. It improves depression by regulating glutamate receptors and inhibiting calcium channels and serotonergic factors, such as SERT. Its antiepileptic mechanism is due to an increase in the inhibitory effect of GABA and potassium current and a decrease in sodium current.

Several animal experiments have suggested the anxiolytic, sedative, analgesic, anticonvulsive, and neuroprotective properties of lavender [157]. Lavender possesses an anticonflict effect in mice [158]. Essential oils obtained from *L. angustifolia* and *R. officinalis* improved cognitive functions and memory in animal models. Lavender oil improved antioxidant enzyme activity, reduced lipid peroxidation, markedly decreased the concentration of the cytokines, tumor necrosis factor-α, and interleukin-1β, and improved migraine in human, animal, and cell studies.

### 4.1 Safety

Lavender is probably safe to consume in the amounts commonly used in foods. Short-term oral intake in the amounts tested in lavender studies on anxiety or other conditions may also be safe. The topical use of products containing lavender may cause allergic skin reactions in some people. A few cases of swelling of breast tissue have been reported in children who used topical products containing lavender. However, it is unclear whether the lavender was responsible for breast swelling, a condition that can have many causes. Little is known about the safety of using lavender during pregnancy or while breastfeeding.

## 5 Conclusion

Published reports clearly have suggested that lavender is an important medicinal plant in traditional medicine. The presence of various compounds, including terpenes, such as linalool, limonene, triterpenes, linalyl acetate, alcohols, ketones, polyphenols, coumarins, cineole, flavonoids, vitamins, and some trace metals are also reported in the seeds of this plant, which add value to its medicinal properties. In conclusion, all these findings strongly support the traditional uses of lavender. More clinical studies are needed to investigate the effectiveness of the plans’ pharmacological active constituents to overcome life-threatening diseases, such as several neurological disorders, including epilepsy, depression, anxiety, migraine, and AD as known diseases of the central and peripheral nervous systems.

## 6 Limitations and futures

The most important limitation of this review is the low average quality of available studies on the topic. The majority of included RCTs were characterized by a high overall risk of bias. Another limitation is the heterogeneity of study designs, especially in non-oral ways of administration. Overall, oral administration of lavender essential oil is effective in the treatment of anxiety, whereas, regarding inhalation, there is only an indication of a reasonable effect, due to the heterogeneity of available studies. Lavender essential oil administered through massage appears effective, but available studies are not sufficient to determine whether this effectiveness is due to a specific effect of lavender. Further high-quality RCTs with more homogeneous study designs are needed to confirm these findings. Available information outlines a safe profile for lavender-based interventions, although more attention should be paid to the collection and reporting of safety data in future studies. Considering these findings, as treatments with lavender essential oil generally seem safe, and, in the case of inhalation, also simple and inexpensive, they are a therapeutic option, which may be considered in some clinical settings. The production of essential oil-based medicines as multi-potent targets is felt highly. Although no side effects have been reported about aromatherapy with lavender on some disorders, more attention should be paid to adverse effects by allergists and healthcare providers. Robust study design, longer follow-up, and larger sample sizes in various studies should be considered in future investigations.

## Author contribution statement

All authors listed have significantly contributed to the development and the writing of this article. All authors read and approved the final manuscript.

## Funding statement

The current study was funded (Grant No.: IR.UMSHA.REC.1400.771) by 10.13039/501100004697Hamadan University of Medical Sciences, Hamadan, Iran.

## Declaration of competing interest

The authors declare the following financial interests/personal relationships which may be considered as potential competing interests:

Alireza Komaki reports was provided by Hamadan University of Medical Sciences. Alireza Komaki reports a relationship with Hamadan University of Medical Sciences that includes: employment.
